# Porcine Epidemic Diarrhea Virus Induces Vero Cell Apoptosis via the p53-PUMA Signaling Pathway

**DOI:** 10.3390/v13071218

**Published:** 2021-06-24

**Authors:** Lin Yang, Chenyu Wang, Jinqi Shu, Huapeng Feng, Yulong He, Jian Chen, Jianhong Shu

**Affiliations:** 1 College of Life Sciences and Medicine, Zhejiang Sci-Tech University, Hangzhou 310018, China; Yanglinhch@163.com (L.Y.); wangchenyu0604@163.com (C.W.); shujq1997@163.com (J.S.); fenghuapeng@zstu.edu.cn (H.F.); heyulong2003@163.com (Y.H.); chj1999@126.com (J.C.); 2 Shaoxing Biomedical Research Institute, Zhejiang Sci-Tech University, Shaoxing 312000, China

**Keywords:** porcine epidemic diarrhea virus, transcriptome sequencing, P53 signaling pathway, Puma, BAX/Bcl-2, Apoptosis

## Abstract

Porcine Epidemic Diarrhea Virus (PEDV) is the causative agent of swine epidemic diarrhea. In order to study the pathogenic mechanism of PEDV, PEDV was inoculated into Vero cells cultured in vitro, and the total RNA of Vero cells was extracted to construct a library for Illumina high-throughput sequencing and screening of differentially expressed genes (*p* < 0.05). Five differentially expressed genes for qRT-PCR verification analysis were randomly selected, and the verification results were consistent with the transcriptome sequencing results. The Kyoto Encyclopedia of Genes and Genomes (KEGG) signal pathway enrichment analysis was performed on the differentially expressed genes screened above. The results showed that the target gene annotations of differentially expressed genes in the African green monkey genome were mainly enriched in the TNF signaling pathway, the P53 signaling pathway, the Jak-STAT signaling pathway, the MAPK signaling pathway, and immune inflammation. In addition, it has been reported that Puma can promote apoptosis and is a key mediator of P53-dependent and non-dependent apoptosis pathways. However, there is no report that PEDV infection can activate Puma and induce apoptosis in a P53-dependent pathway. It was found by flow cytometry that PEDV infection induced apoptosis, and by Western Blotting detection, PEDV infection significantly increased the expression of p53, BAX, and Puma apoptosis-related proteins. Treatment Vero cells with the p53 inhibitor, PFT-α, could significantly inhibit PEDV-induced apoptosis. Studies have shown that PEDV infection can activate Puma and induce apoptosis in a P53-dependent pathway. These findings provide data support for further elucidating the pathogenic mechanism of PEDV and developing an effective vaccine against PEDV.

## 1. Introduction

PEDV belongs to the genus Nidovirales, Coronaviridae, Alpha Coronavirus, and is the causative agent of swine epidemic diarrhea [1,2]. PEDV infection mainly causes severe diarrhea, vomiting, and dehydration in pigs, resulting in extremely high mortality. The mortality rate of piglets within 6 days of being infected by PEDV is up to 100% [3,4]. Since the disease first broke out in the United Kingdom in 1971, it has been endemic in many countries including Asia and Europe [5]. At present, PEDV has broken through the regional spread and spread across the world, resulting in tremendous economic loss for global pig industry [3]. There is no effective control method for PEDV [6]. Therefore, the basic research on the pathogenic mechanism of PEDV is significantly important. There have been a large number of reports about PEDV infecting Vero cells in vitro [7,8], such as transcriptome [9], proteome [10], etc., but the study on repeating sequence analysis of Vero cells after PEDV infection at different times has not been reported. In this study, Illumina transcriptome sequencing technology was used to analyze Vero cells at different times after PEDV infection. The data based on transcriptome sequencing technology can be used to explore differentially expressed genes and related regulatory signaling pathways involved in PEDV infection after Vero cells, which contribute to investigating the pathogenesis of PEDV and providing data for vaccine development.

The tumor-suppressor protein 53 (p53) is a sequence-specific DNA-binding protein that plays the role of a transcription factor to regulate cellular processes such as cell cycle arrest, senescence, and apoptosis [11]. Studies have reported that the H3N2 canine influenza virus that infects MDCK cells and the novel H7N9 influenza A virus that infects A549 cells can induce apoptosis by activating p53 [12,13]. Under normal circumstances, p53 can be stabilized at low levels, such as in MDM2, which acts as a ubiquitin ligase to assist p53 degradation, and these processes will change when cellular stress responds to external stimuli. Activated and phosphorylated p53 translocate to the nucleus to regulate transcription [14].

Puma is expressed at a low level in normal cells, but when the cells are stimulated in a stressed state, Puma is quickly transcribed and translated, resulting in increasing expression efficiently and promoting apoptosis [15]. This was also verified in the results of this transcriptome sequencing. The expression level of Puma in normal Vero cells was extremely low, but after PEDV infection for 24 h, the expression level of Puma significantly increased. Puma is a member of the BH3-only subfamily of the Bcl-2 family. Yu et al. [15] and Nakanoo et al. [16] analyzed gene expression profiles in two laboratories in 2001 and found that Puma is a target gene downstream of p53, it is p53-dependent, and is a key mediator dependent on the apoptosis pathway [16,17,18].

In this study, Illumina transcriptome sequencing technology was used to analyze Vero cells after PEDV infection at different times, and the differentially expressed genes and related regulatory signaling pathways involved in PEDV-infected Vero cells were screened. The transcriptome sequencing data showed that the expression level of p53 and Puma genes was significantly upregulated after being infected by PEDV for 24 h. It is speculated that PEDV infection may be induced by Puma and induce apoptosis in a p53-dependent pathway. The experiments have shown that PEDV infection can activate Puma and induce apoptosis in a P53-dependent pathway. These findings provide data support for further elucidating the pathogenic mechanism of PEDV and developing an effective vaccine against PEDV.

## 2. Materials and Methods

### 2.1. Cell Culture and PEDV Infection

Vero cells and the porcine epidemic diarrhea virus are preserved by the College of Life Sciences and Medicine, Zhejiang Sci-Tech University.

Vero cells were cultured in Dulbecco’s Modified Eagle Medium (DMEM; Gibco, Grand Island, NE, USA) supplemented with 10% heat-inactivated fetal bovine serum (FBS; Gibco, Grand Island, NE, USA), 1% penicillin/streptomycin (Invitrogen, Carlsbad, CA, USA) at 37 °C in a 5% CO_2_ atmosphere in an incubator. Vero cells infected with PEDV usually induce typical cytopathic effect (CPE). Vero cells were cultured in a 6-well cell culture plate. PEDV was inoculated in Vero at 0.5 MOI when the cells grew to 95% confluence, then cultured for 12, 24, 36, and 48 h. The morphological changes of PEDV-infected Vero cells were observed to determine the sample conditions for transcriptome sequencing.

### 2.2. Total RNA Extraction and Quality Inspection of Vero Cell

Total RNA was extracted by the Trizol reagent (Invitrogen, Carlsbad, CA, USA) on the basis of the manufacturer’s protocol. The concentration and purity of total RNA were assessed by Nanodrop (Thermo Fisher Scientific, Waltham, MA, USA), and the integrity of total RNA was detected by agarose gel electrophoresis.

### 2.3. cDNA Library Construction and High-Throughput Sequencing

The total RNA of the three groups of 9 samples (uninfected with PEDV, infected with PEDV for 12 h, infected with PEDV for 24 h) was sent to Majorbio Technology Co., Ltd. (Shanghai, China) for transcriptome sequencing analysis. First, the library construction was performed using the Illumina TruseqTM RNA sample prep Kit (Illumina, San Diego, CA, USA) method. After the library was qualified, 9 samples were sequenced by Illumina high-throughput sequencing technology.

### 2.4. Reverse Transcription-Quantitative Polymerase Chain Reaction (RT-qPCR) Analysis

KEGG enrichment analysis showed that CDKN1A, MDM2, FAS, CASP3, CDK1, puma, CCNB1, and other genes were enriched in the p53 signaling pathway. In order to confirm the result of transcriptome sequencing, 5 genes with higher expression levels above were randomly selected for real-time quantitative PCR. The above five genes’ primers were designed by using Primer select software in DNAStar software and referring to the principle of designing qRT-PCR primers, as shown in Table 1. β-actin was used as the house-keeping gene for internal normalization for qRT-PCR experiments. The designed primer sequences of the 6 groups of genes were sent to Sangon Biotech Co., Ltd. (Shanghai, China) for synthesis.

For RT-qPcR analysis, the Prime Script Reagent kit (Takara Bio, Inc., Otsu, Japan) was used for cDNA synthesis in a reverse transcription reaction. The standard sample reaction system is: 5 × PrimerScript Buffer (for Real Time) 4 μL; PrimerScript RT Enzyme Mix Ι 1 μL; RT Primer Mix 1 μL; total RNA 300 ng; and add RNase Free ddH2O to a total volume of 10 μL. The reaction was incubated at 37 °C for 15 min and at 85 °C for 5 s and then amplified by gene-specific primers for qPCR.

The qPCR analysis was set up with SYBR Premix Ex Taq (Takara Bio, Inc., Otsu, Japan) and performed using the 7500 Real-Time PCR system (Applied Biosystems; Thermo Fisher Scientific, Inc.). The qPCR reaction system is: SYBR® Premix Ex TaqTM II (2×) 10 μL; ROX Reference Dye II (50×) 0.4 μL; PCR Forward Primer (10 μM) 0.8 μL; PCR Reverse Primer (10 μM) 0.8 μL; cDNA 2.0 μL; and add RNase Free ddH2O to a total volume of 10 μL. After initial denaturation at 95 °C for 30 s, cDNA was amplified in 40 cycles. Amplification conditions were: 3 s of denaturation at 95 °C, 30 s of annealing at 60 °C, and 30 s of extension at 72 °C, followed by a final extension step at 72 °C for 10 min. The experimental data are analyzed using the 2−ΔΔCT method, and β-actin was used as the house-keeping gene for internal normalization.

### 2.5. GO Function Annotation Analysis of Differentially Expressed Genes

The differentially expressed genes were classified and annotated according to the biological process (BP), molecular function (MF), and cellular component (CC) by the GO database. Through GO annotation, we can understand the functional classification of differentially expressed genes.

### 2.6. KEGG Enrichment Analysis of Differentially Expressed Genes

R language was used to write script to analyze KEGG pathway enrichment of genes in the gene set. When the *p* value (*p* value_uncorrected) < 0.05, it was considered to be significant.

### 2.7. Apoptotic Rate Measurement

Cell apoptosis was measured according to the manufacturer’s protocol with the FITC Annexin V Apoptosis detection kit (Becton Dickinson Biosciences, Franklin Lakes, NJ, USA). Briefly, cells were rinsed twice with PBS and re-suspended in 100 μL binding buffer, followed by the addition of 5 μL of Annexin V-FITC and 5 μL of PI. After incubation in the dark for 15 min at 37 °C, 400 μL of the binding buffer was added. Moreover, 10,000 cells were acquired, and a percentage of positive cells was analyzed by flow cytometry (Becton Dickinson).

### 2.8. Western Blotting Analysis

The cells were washed twice with PBS, then treated with RIPA Lysis Buffer with 1 mM phenylmethanesulfonyl fluoride (PMSF). Protein concentrations were measured by BCA Protein Assay Reagent. Equal quantities of the total protein (40 µg) were separated on a 12% SDS-PAGE gel at 120 V for 1 h. Subsequently, proteins were transferred to polyvinylidene difluoride (PVDF) membranes (Millipore Corp., Atlanta, GA, USA) at 300 mA for 90 min at 4 °C. The membranes were blocked in Tris-buffered saline with 0.1% Tween 20 (TBST) containing 5% skimmed milk powder at room temperature for 2 h, and then incubated with the indicated primary antibodies dissolved in bovine serum albumin (BSA) overnight at 4 °C, followed by culturation with HRP-conjugated secondary antibodies at room temperature for 1 h. The signal was detected using the ECL reagent (GE Healthcare).

### 2.9. MTT Treatments

Pifithrin-α can reversibly inhibit p53-dependent gene transcription activation, such as cyclin G, p21/waf1, and MDM2 protein. Pifithrin-α not only inhibits p53-mediated apoptosis induced by cytotoxins, but also inhibits p53-mediated cell apoptosis [19]. MTT assay was used to exclude the effect of PFT-α on cell activity (Roche Applied Science). Vero cells plated into 96-well plates, at around 90% confluence, were treated with a corresponding concentration of the inhibitor and incubated for 24 h, then 10 μL of MTT at a concentration of 5 mg/mL was added to each well and incubated at 37 °C for 4 h. Subsequently, 100 μL of DMSO was added and the absorbance at 490 nm was measured in a Micro plate reader.

### 2.10. Inhibitor Treatments

Pifithrin-α (PFT-α) was stored as a 10 mM stock solution in DMSO and was diluted to the working concentration of 10 μM with the cell maintenance medium. When Vero cells were treated with PFT-α, the supernatant was discarded, washed 3 times with PBS, 1 mL of PFT-α was added to each well, and the negative control and blank control were set up, pretreated for 1 h, and then inoculated with 1 MOI of PEDV. After virus adsorption for 90 min, the virus solution was removed, washed twice with PBS, 1 mL of PFT-α was added, and it was placed in a 5% CO_2_ atmosphere at 37 °C in an incubator for 24 h. Different treatments were carried out according to the needs of the experiment.

## 3. Results

### 3.1. PEDV Infection Induced the Pathological Effect of Vero Cells at Different Time Points

Inverted fluorescence microscopy was used to observe the cytopathic effect at 12, 24, 36, and 48 h after Vero cells were inoculated with PEDV (MOI = 0.5). It showed that the cells in the uninfected control group had no cytopathic effect. When infected with PEDV for 12 h, the cells showed little change compared with the uninfected group. After being infected with PEDV for 24 h, some cells presented severe pathological effects, but the cells were not broken and shed. The cells infected with PEDV for 36 h presented a considerable pathological effect, and were also broken and shed. When infected for 48 h, 90% of Vero cells presented a pathological effect and were accompanied by cell breakage and shedding. Therefore, in this study, the cell samples of the uninfected control group, the 12 h infected group, and the 24 h infected group were selected as analysis samples for subsequent transcriptome sequencing, and the uninfected group was used as the control.

### 3.2. Quality Test of Total RNA

**Figure 1:**
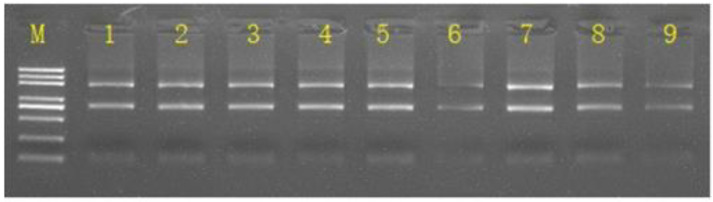
The gel electrophoresis diagram of total RNA from Vero cell. M: Marker; Line 1–3: The uninfected control group; Line 4–6: Infected with PEDV for 12 h; Line 7–9: Infected with PEDV for 24 h.

The concentration and purity of the extracted total RNA were detected by Nanodrop. The results showed that the concentration of total RNA extracted from the three sets of samples to be sequenced was ≥50 ng/μL, and the OD 260/280 was 1.8–2.2. Gel electrophoresis detection showed clear 28 S rRNA and 18 S rRNA bands, as shown in Figure 1. This indicates that the total RNA extracted from each group of cells has good integrity and can be used for subsequent library construction and sequencing.

### 3.3. Analysis of Differentially Expressed Genes in Infected Vero Cells Compared to the Uninfected Cells

Illumina high-throughput sequencing technology was used to sequence the three groups of samples, and DESeq2 software was performed to identify genes that were differentially expressed between groups. Compared with the uninfected control, there were four differentially expressed genes in the group infected with PEDV for 12 h, including one up-regulated gene and three down-regulated genes (*p* < 0.05). Meanwhile, 1498 differentially expressed genes were showed in the group infected with PEDV for 24 h, of which 956 genes were upregulated and 542 genes were downregulated (*p* < 0.05). Compared with the group infected with PEDV for 12 h, there were 1643 differentially expressed genes in the group infected with PEDV for 24 h, including 1079 upregulated genes and 564 downregulated genes (*p* < 0.05) (Table 2). The discovery of these differentially expressed genes laid the foundation for finding specific target genes.

### 3.4. qRT-PCR Verification of 5 Seleted Differentially Expressed Genes

**Figure 2:**
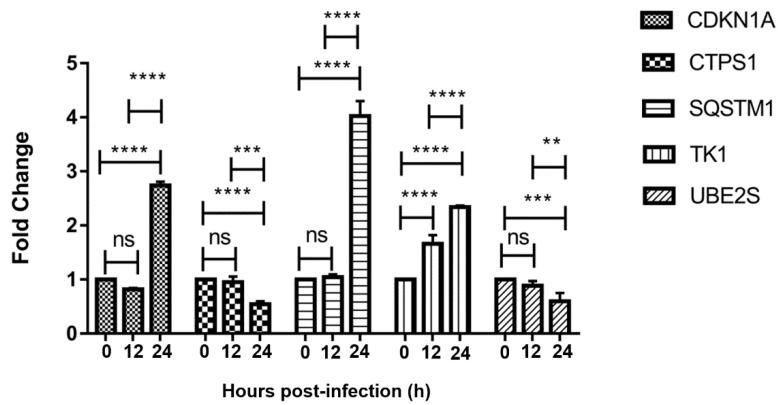
qRT-PCR verification of CDKN1A, CTPS1, SQSTM1, TK1, and UBE2S. ns, not significant; **, *p* < 0.01; ***, *p* < 0.001; ****, *p* < 0.0001.

The relative expression of five genes (CDKN1A, CTPS1, SQSTM1, TK1, UBE2S) in Vero cells uninfected with PEDV, infected with PEDV for 12 h, and for 24 h was verified by qRT-PCR (Figure 2). The five genes were stably expressed in Vero cells and the trend of the expression level was consistent with the sequencing results.

### 3.5. Analysis of GO Function Annotation of Differentially Expressed Genes

**Figure 3:**
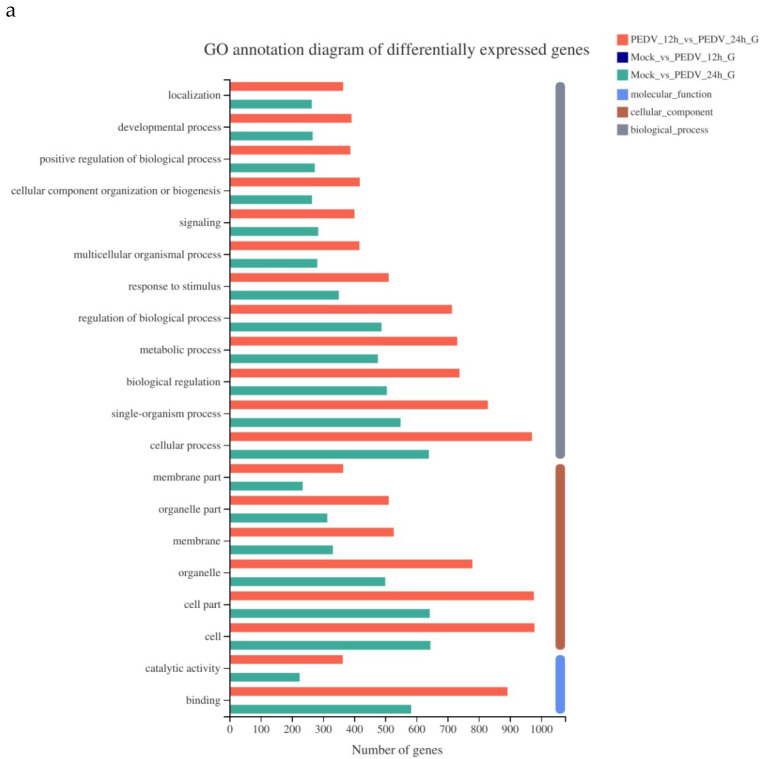

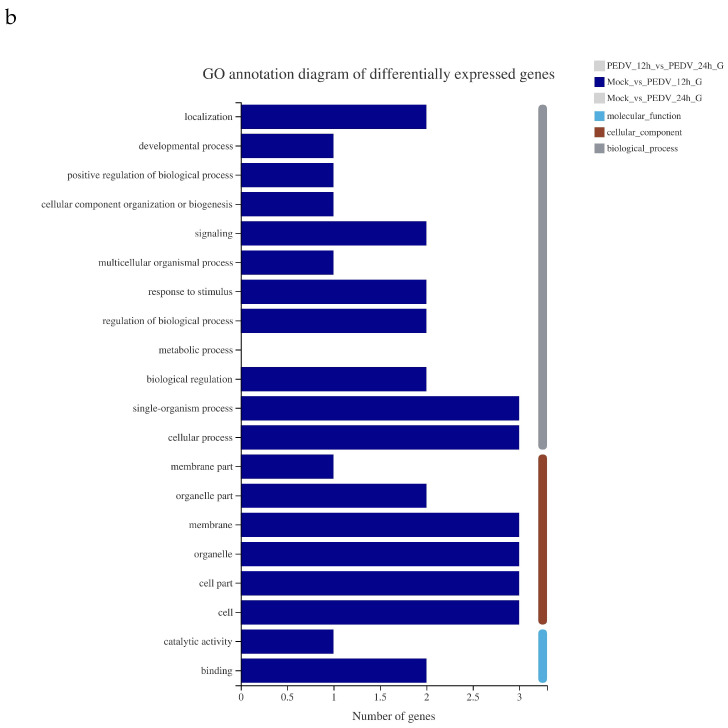
GO function annotation of differentially expressed genes of PEDV 12 h vs. PEDV 24 h and Mock vs. 24h (**a**) Mock vs. 12 h (**b**).

As shown in Figure 3, the GO function annotation analysis of the differentially expressed genes involves three aspects: Biological process, cell composition, and molecular function, and involves cellular process, biological regulation, metabolic process, cellular composition, organelle composition, and catalytic activity. Due to the large abscissa value in Figure 3a, the GO function annotation of differentially expressed genes of Mock vs. 12 h is listed separately Figure 3b.

### 3.6. KEGG Enrichment Analysis of Differentially Expressed Genes

**Figure 4:**
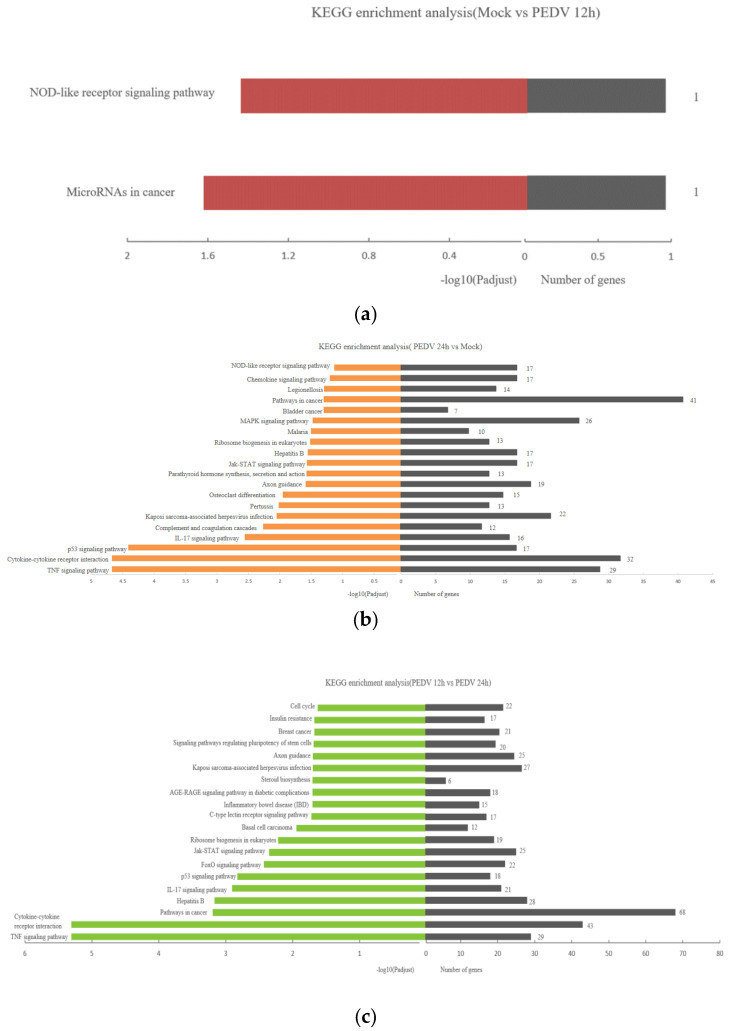
KEGG enrichment map of differentially expressed genes of Mock vs. PEDV 12 h (**a**) Mock vs. PEDV 24 h (**b**) and PEDV 12 h vs. PEDV 24 h (**c**).

The KEGG enrichment analysis of the differentially expressed genes screened above is shown in Figure 4. The smaller the FDR and the larger the −log10 (FDR) value, the more significantly the KEGG pathway is enriched. Compared with the mock, where the Vero cells infect PEDV for 12 h, the target gene annotations of four differentially expressed genes in the African green monkey genome were only enriched in the NOD-like receptor signaling pathway and microRNAs in the cancer signaling pathway. After Vero cells were infected for 24 h, the target gene annotations of differentially expressed genes in the African green monkey genome are mainly enriched in the TNF signaling pathway, the p53 signaling pathway, the Jak-STAT signaling pathway, the MAPK signaling pathway, and immune inflammation. Vero cells infected for 24 h were compared with Vero cells infected for 12 h, and showed that the TNF signaling pathway and pathways in cancer are significantly enriched.

### 3.7. PEDV Infection Induces Apoptosis in a Dose-Dependent Manner

**Figure 5:**
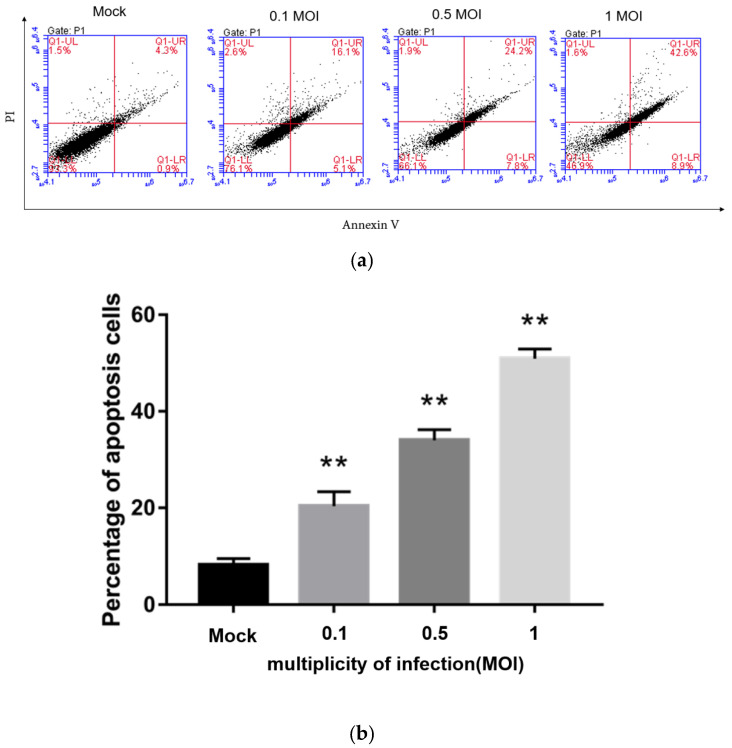
Detection of Vero cell apoptosis rate after PEDV infection by flow cytometry at different MOIs by Flow cytometry (**a**) and the percentage of apoptosis cells (**b**). **, *p* < 0.01.

Flow cytometry was used to detect the apoptosis rate of Vero cells infected with PEDV at 0, 0.1, 0.5, and 1 MOI by Annexin-PI double staining at 24 h post-infection. The results are shown in Figure 5. Compared with the non-infected PEDV group, the groups infected with PEDV at different MOIs all showed different degrees of apoptosis, and the higher the dose that Vero cells were infected with, the more apoptosis, indicating that PEDV infection induces Vero cell apoptosis in a dose-dependent manner. When Vero cells were infected with 1 MOI PEDV, the apoptosis rate reached about 50%.

### 3.8. PEDV Infection Affects the Expression Level of Apoptosis-Related Proteins

**Figure 6:**
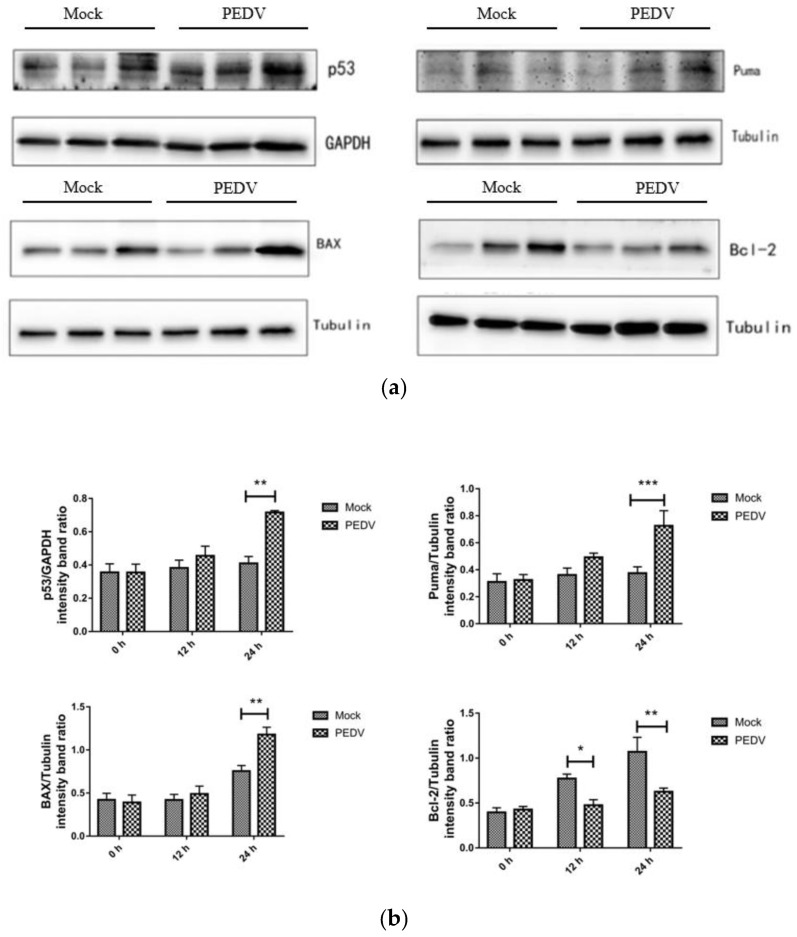
Effect of PEDV infection on p53 signaling pathway-related protein expression by Western blotting (**a**) and the intensity band ratio of Western blotting (**b**). *, *p* < 0.05; **, *p* < 0.01; ***, *p* < 0.001.

Western blotting was used to detect the expression of apoptosis-related proteins at different times (0 h, 12 h, 24 h) in Vero cells that were not infected with PEDV and infected with PEDV. As shown in Figure 6, compared with the uninfected group, the expression levels of P53, Puma, BAX, and Bcl-2 did not change significantly when Vero cells were infected with PEDV for 12 h (*p* > 0.05). When Vero cells were infected with PEDV for 24 h, the protein expression of P53, Puma, and BAX increased significantly, and the protein expression of Bcl-2 decreased significantly.

### 3.9. The Effect of Inhibitor PFT-α on Proteins of p53, Puma, BAX and Bcl-2

**Figure 7:**
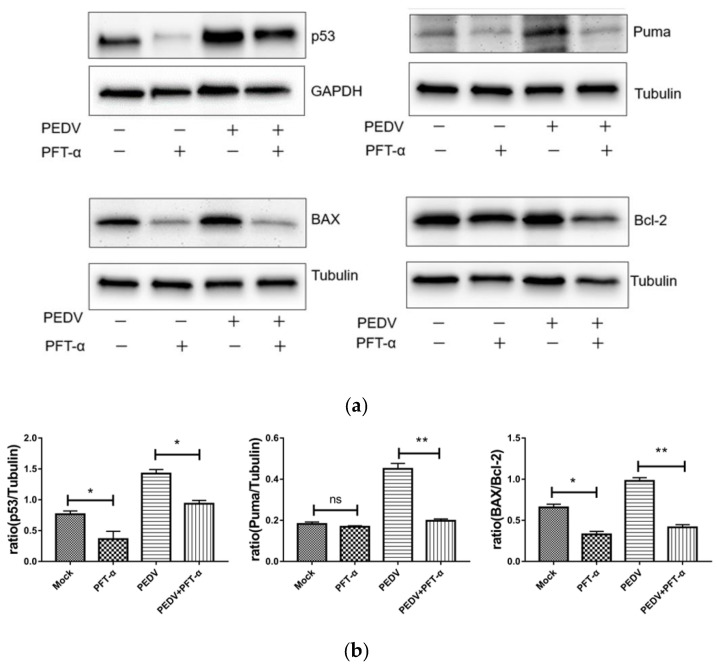
Effect of inhibitor PFT-α on proteins of p53, Puma, BAX, and Bcl-2 by Western blotting (**a**) and the intensity band ratio of Western blotting (**b**). ns, not significant; *, *p* < 0.05; **, *p* < 0.01.

Western blotting was used to detect the changes of proteins p53, Puma, BAX, and Bcl-2 under PFT-α (10 mM) treatment for 24 h. As shown in Figure 7, PEDV infection can significantly promote the expression of p53 and Puma, and upregulate the ratio of BAX/Bcl-2. Treatment with PFT-α can significantly reduce the expression of p53 and Puma, and downregulate the ratio of BAX/Bcl-2, and the downregulated ratios of Puma and BAX/Bcl-2 indicate that inhibition of p53 can significantly reverse the apoptosis induced by PEDV.

### 3.10. The Inhibitor PFT-α Treatment Inhibits PEDV-Induced Apoptosis with no Effect on Cell Viability

**Figure 8:**
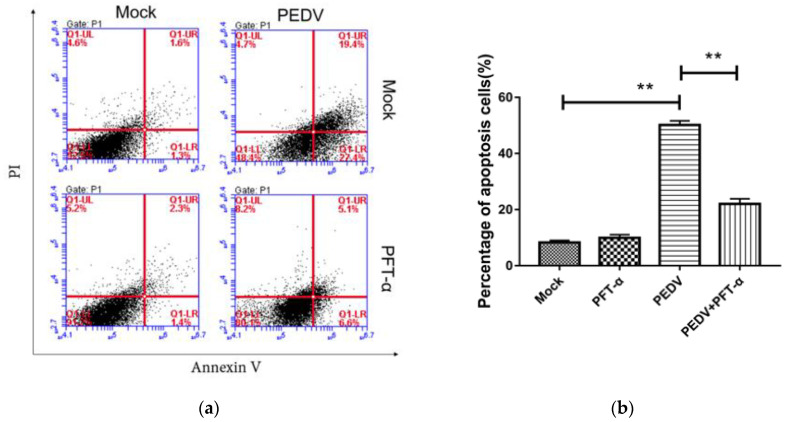
Detection of PFT by MTT- α Inhibition of cell activity (**a**) and percentage of apoptosis cells (**b**). **, *p* < 0.01.

**Figure 9:**
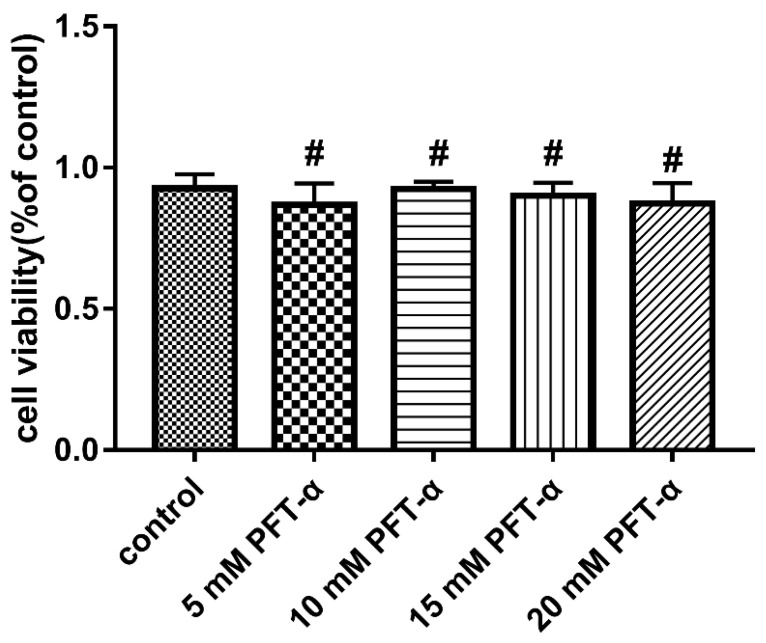
Effects of different concentrations of PFT-α treatment on cell viability (#, *p* < 0.05).

Flow cytometry was used to detect the apoptosis rate of each experimental group under the treatment of PFT-α (10 mM) for 24 h. As shown in Figure 8, compared with the PEDV-uninfected group, PEDV infection significantly increased the apoptosis rate. Compared with the PEDV-infected group, PFT-α treatment significantly reduced the PEDV-induced apoptosis rate. In order to exclude the effect of the inhibitor of PFT-α on cell viability, the MTT method was used to detect the inhibitory effect of PFT-α on cell activity. As shown in Figure 9, different concentrations of PFT-α (5 mM, 10 mM, 15 mM, 20 mM)-treated cells for 24 h did not affect the cell viability.

## 4. Discussion

Although porcine epidemic diarrhea only causes mild symptoms such as weight loss and low mortality when infecting adult pigs, it shows a high incidence of 100% and a high mortality rate up to 90–100% when it infects newborn piglet [20]. PEDV has been reported to be prevalent in the world, causing huge economic losses to the swine industry all over the world and seriously affecting the development of the world’s pig industry. Therefore, basic research on PEDV is particularly important.

Analysis of transcriptome sequencing data showed that when PEDV infected the Vero cells for 12 h, there were only four differentially expressed genes, of which the MDGA1 gene was significantly upregulated, and GBP2, MN1, and BMF genes were significantly downregulated. However, when PEDV infected the Vero cells for 24 h, the GBP2 gene was significantly upregulated (*p* < 0.05). 

The cyclin-dependent kinase inhibitor 1A (CDKN1A) is a cell cycle inhibitor, directly controlled by p53-dependent or independent pathways, and is involved in terminal differentiation, stem cell renewal, apoptosis, and cell migration [21,22]. However, the specific role and mechanism of CDKN1A in PEDV are still unclear.

GBP-2 belongs to a family of 65–67 kDa GTPases, the guanylate binding proteins (GBP), induced by IFNs. GBP genes have been used as markers for IFN responsiveness in both cells and organisms because they are some of the most highly expressed genes after IFN-γ stimuli, being induced within a few minutes to transcription induction by IFN-γ. However, to date, the biological function of GBP-2 has received little attention, and there are no data on its involvement in PEDV. It has been previously reported that the expression of GBP-2 gene continues to increase in a p53-dependent manner, and RNA interference inhibits p53 expression to confirm this. Previous results suggest that GBP-2 is not a conventional transcriptional target of p53 but that its effect may be related to the capacity of cells to maintain a sustained proliferative and survival capacity. Thus, GBP-2 is upregulated in a p53-dependent manner through a mechanism that may involve complex formation between the wild-type form of p53 and the transcription factor IRF-1 (interferon regulatory factor-1) [23]. Upregulation of the GBP2 demonstrated that GBP2 is an important candidate gene for disease resistance. In the gene expression profile analysis of Salmonella choleraesuis infection, it was found that both GBP1 and GBP2 of pigs were significantly upregulated by nearly 10 times [24]. Current studies on the GBPs family have confirmed that it has an antiviral effect, but the underlying mechanism is not yet clear [25,26]. Studies have shown that high expression of porcine GBP1 and GBP2 genes can inhibit the proliferation of porcine reproductive and respiratory syndrome virus (PRRSV) and pseudorabies virus (PRV), but whether high expression of GBP1 and GBP2 genes can inhibit the PEDV replication in Vero cells needs further study.

**Figure 10:**
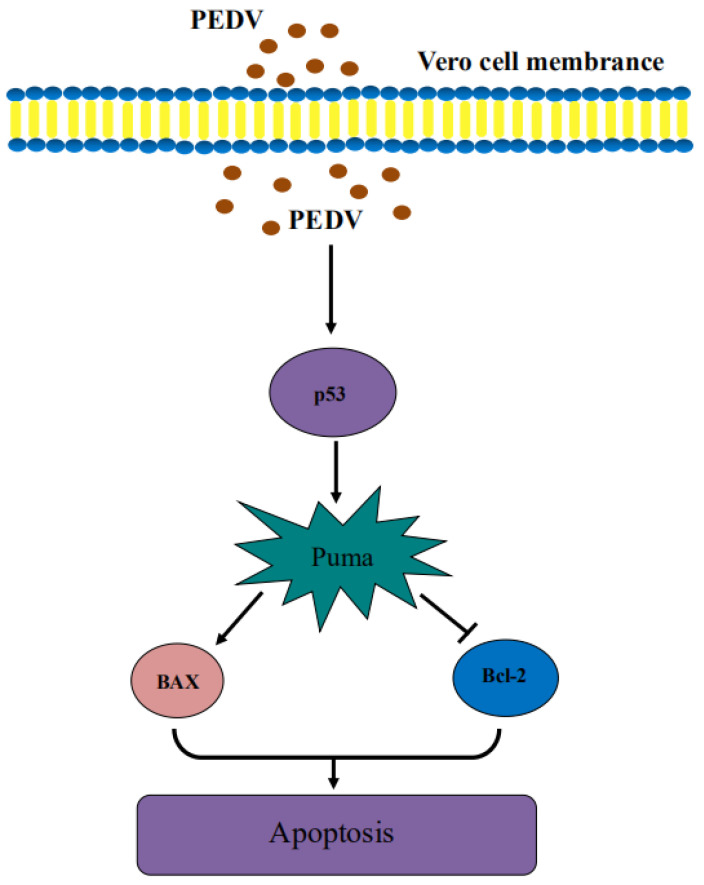
Schematic diagram of PEDV activating Puma and inducing apoptosis in a p53-dependent pathway.

Studies have shown that apoptosis occurs in cells after PEDV infection [8]. Among the differentially expressed genes screened in this study, there are also differential expression of apoptosis-related genes, such as p53, BAX, Bcl-2, Puma, etc. It is worth noting that the Puma gene is a member of the BH3-only subfamily of the Bcl-2 family, which can promote cell apoptosis and is a key mediator of p53-dependent and independent apoptosis pathways. More and more studies have shown that Puma is a key mediator of apoptosis. Based on the significant upregulation of the expression of P53 and Puma genes when PEDV infects Vero cells for 24 h, it is speculated that PEDV infection may be caused by activating Puma and inducing apoptosis through a p53-dependent pathway. Above all, as shown in Figure 10, we found that PEDV infection may activate Puma and induce apoptosis in a p53-dependent pathway in this study.

## Figures and Tables

**Table 1:** Oligonucleotide primers used for RT-qPCR.

Primer	Primer Sequence (5′ to 3′)
β-actin F	CTTCCTGGGTGAGTGGAGAC
β-actin R	GAAGGTAGTTTCATGGATGCC
CDKN1A F	CAGGAGGCTCGTGAACGATG
CDKN1A R	CCTGTGGGAAGGTAGAGCTTG
CTPS1 F	CAAGGAACGGAAAGGAGATTACTTGG
CTPS1 R	CACGGTTCCACCAAGCTCAATAAC
SQSTM1 F	CCTTCGGGCACCTGTCTGAG
SQSTM1 R	GATGGACCAGCAGCTGATTC
TK1 F	CCTTCGGGCACCTGTCTGAG
TK1 R	GATGGACCAGCAGCTGATTC

**Table 2:** Numbers of differentially expressed genes (DEGs).

	All DEGs	Down-Regulated Genes	Up-Regulated Genes
Mock vs. PEDV 12 h	4	1	3
Mock vs. PEDV 24 h	1498	956	542
PEDV 12 h vs. PEDV 24 h	1643	1079	564

## Data Availability

The data that support the findings of this study are available on request from the corresponding author. The data are not publicly available due to privacy or ethical restrictions.
